# The Second Annual Report

**Published:** 1914-08-08

**Authors:** 


### NATIONAL INSURANCE ACT DEVELOPMENTS.

### The Second Annual Report.

A voluminous report,* extending to nearly six hundred pages, has recently been issued, giving an account of the administration of national health insurance for the year 1913-1914. The work is similar in character and design to the report which was issued early in July 1913, and it appears from the introduction that it is the intention of the Government, as represented by the Insurance Commissioners, to make the report an annual production. To those who have taken a watchful interest in the developments of the system of national health insurance the perusal of the contents of the report will present much that is already familiar. But even if the book contained no more than a simple record of the numerous orders, regulations, and instructions that have been issued from time to time by the Insurance Commissioners, it would still serve a useful purpose in bringing them together in a single volume. The report is, however, much more than a simple record of regulations and orders, and a great deal of the material is absolutely fresh. It is not the least welcome feature of the report that the information contained therein is, on the whole, so closely up to date. The Insurance Commissioners are to be congratulated on the issue early in July last of a work which contains information on certain aspects of the administration of the Acts as closely up to date as the beginning of May.

### National Insurance and Public Health.

The report is divided into six main sections, and these are followed by five appendices in which are contained numerous statistical tables by means of which the progress of the administration of the Acts may be measured. Part I. is a short introduction, in which the scope of National Health Insurance is defined, and wherein is also set forth the principal aims and objects of the Amendment Act of 1913, with short comments on the effects of the alterations made by that enactment. It has come to be recognised as the result of experience that National Health Insurance is by no means confined to the mere administration of the machinery brought into operation by the Acts. It has been found by those responsible for the administration of the Acts that they have had to have constant recourse to information and advice from authorities and bodies working in most diverse fields, and, reciprocally, they are themselves constantly approached by such authorities and bodies seeking to secure the fruitful application of the provisions of the National Insurance Acts to the particular social activity in which they are interested.

The types of question with which National Health Insurance, in its full development, is most concerned are those which relate to public health and to economic conditions.

It is pointed out that in the sphere of public health there are boundless opportunities for co-operation between National Health Insurance and Local Government. This is emphasised in the report itself by reference to the part that has been played by the local authorities and voluntary bodies in conjunction with the central insurance authorities in the administration of sanatorium benefit.

### The Work of the Voluntary Hospitals.

It is also particularly interesting and important to those who are concerned in the administration of the voluntary hospitals to note the remarks set forth in the report on the subject of medical benefit. "The working of medical benefit has already revealed, more clearly than had previously been possible, the loss which the country has suffered through the past inability of large sections of the community to obtain adequate treatment of the diseases from which they suffered. It is true that in certain areas, and for certain types of disease, the great voluntary hospitals made readily available, for even the poorest of the population, the most perfect forms of treatment known to science; but the greatest services performed by the hospitals have been in connection with illnesses and accidents forming suitable cases for institutional treatment. The work of the out-patients' departments is necessarily limited; and, further, there are large areas where there are no hospitals treating persons otherwise than as residential inmates; practically throughout the country there has existed no organisation of the provision of medical treatment for the mass of illnesses which require to be treated in the patient's home." It is claimed that the institution of medical benefit has not only provided the means for this home treatment, but has also laid "a foundation on which extended provision can suitably be based." In view of these remarks one is tempted to ask if the true value of the hospitals in the treatment of really serious illnesses has been appreciated, and if the co-operation, which the Insurance Commissioners so heartily desire, is to be reciprocated by any financial assistance, without which the hope of extended provision appears to be but an idle dream.

### Economic Aspects of National Insurance.

In connection with the economic aspects of the questions relating to National Health Insurance, the Commissioners look to the payment of sickness benefit as affording a means to enable persons to forgo wages while under treatment. It frequently happened in the past that persons could not afford to stay away from their work in order to submit to medical treatment, even though that treatment might have been free, and in consequence the provision of free treatment was often abortive. But since the Insurance Act made provision both for supplying medical treatment and cash payments during illness the workers have been enabled to take

* Cd. 7496. Government Publishers. 2s. 5d.

advantage of the provision. One result of this dual provision, it is claimed, has been to encourage doctors to settle in areas where, owing to the absence of other doctors resulting from the conditions prevalent before the days of National Health Insurance, they would be likely to experience a minimum of competition.

Another economic result of a similar character claimed for the operation of the Act is that persons have been enabled to take advantage of hospital treatment that they would otherwise have been unable to do. The payment of the sickness benefit to the dependants while the insured person is in hospital or sanatorium has enabled such dependants to live while the breadwinner was being restored to health.

It is curious to observe, however, that the introduction to the report entirely omits any reference to the legislation by the Act of 1913 of the payment of sickness benefit to persons who have no dependants in a lump sum on leaving hospital! Although it may be readily admitted that the payment of sickness benefits to dependants while the insured person is in hospital is economically sound in principle, it will hardly be denied that there is no justification for the payment of benefit to be reserved for the use of the insured person himself when he has derived all the advantages that he can from free hospital treatment. And the Insurance Commissioners do not attempt to justify the procedure.

While dealing with this subject it may be remarked that in a later section of the report the English Commissioners refer to the circular which they issued with regard to subscriptions by approved societies to hospitals, dispensaries, nursing associations, etc. After pointing out that they had advised the societies that no special fund or actuarial margin existed for the purpose, and that the benefit funds would have to bear the cost if thought to be justified, they go on to report that "up to the present only a small number of societies have incurred expenditure of this description, the general tendency being, as it would appear, to reserve consideration of the question in order to obtain further experience of the incidence of their ordinary benefit expenditure before deciding to make any commitments of the kind in question."

With regard to agreements between approved societies and hospitals of which members of the Society are inmates, the English Commissioners point out that they have made it clear to both hospital authorities and societies that such agreements must be made before the member enters the institution, or during his stay therein, otherwise no claim for payment can be entertained by a society after the member has quitted the institution. They then recite the provision of the Amendment Act referred to above, but make no comment thereon.

### Medical Research.

Part II. of the report gives an account of the principal activities of the Joint Committee of the Insurance Commissioners during the year. This Committee is concerned with matters in which uniformity of action by the respective Commissions is either imperative or desirable. It is, therefore, to this Committee that the reports of the actuaries are furnished, and it was under their instructions that the regulations were drafted for the purpose of medical research. The promulgation of the scheme for medical research, for which during 1914 the sum of about £55,000 is available, this sum increasing gradually from year to year, must indeed be considered as one of the most important pieces of work that had to be chronicled. Under the scheme that has been devised two separate bodies have been brought into being. The first is known as the Medical Research Committee, the members of which serve in a personal capacity, and their duty is to frame schemes for research. These schemes are referred in turn to the second body, known as the Advisory Council for Research, whose members are representative of scientific interests, and only with their consent and after the approval of the Chairman of the Joint Committee has been obtained can the scheme operate.

It is added that a preliminary scheme has already been submitted and approved, and the work is now in progress. It is to be noted also that the application of the Research Fund is not limited to tuberculosis, but that the money may be expended on research into any disease to which insured persons are subject.

### The Financial Position of Approved Societies.

The remarks under this section of the report on the financial position of approved societies are worthy of note, and the conclusions may be briefly summarised as follows:—

1. The sickness claims of men, taken as a whole, have been within the actuarial provision. 2. The sickness claims of women, taken as a whole, have been materially in excess of that provision. 3. Great variations have arisen between the sickness claims made upon individual societies; while in many cases the claims have exceeded the estimates, a large number of societies may be expected to show surpluses at the first valuation. 4. The sickness claims of both men and women have been in many societies above the standard which should normally prevail, and which may be expected to obtain, when the societies have become experienced in the administration of the Act. 5. The claims for maternity benefit have also varied considerably as between different societies. These conclusions go to confirm the observations we have made from our own investigations, and they are, so far as we know, the first official pronouncement on a very important aspect of the future of National Health Insurance.

Space forbids the treatment of the remaining sections of the report, but it may be stated that they deal with the administration of the Act by the four separate Insurance Commissions in England, Scotland, Ireland, and Wales respectively. Each report, while traversing very much the same ground, has its own special features of interest, and the statistical tables which appear in the appendices give indications of the varying problems that have to be met in the different countries of the Union.

